# Subcutaneous Furosemide Therapy for Chronic Management of Refractory Congestive Heart Failure in Dogs and Cats

**DOI:** 10.3390/ani15030358

**Published:** 2025-01-26

**Authors:** Sergio F. Lombardo, Heidi Ferasin, Luca Ferasin

**Affiliations:** 1 The Ralph Veterinary Referral Centre, Marlow SL7 1YG, UK; sergio@cardiospecialist.co.uk; 2 Specialist Veterinary Cardiology Consultancy Ltd., Four Marks GU34 5AA, UK; heidi@cardiospecialist.co.uk

**Keywords:** loop diuretic, furosemide, torasemide, diuretic resistance, diuretic efficacy, congestive heart failure

## Abstract

Reduced efficacy of oral diuretics in the treatment of congestive heart failure (CHF) can be secondary to reduced enteral drug absorption. The aim of this study was to determine the efficacy of subcutaneous (SC) furosemide administration to control the signs of refractory CHF in dogs and cats and to assess the feasibility of this route of administration. The clinical records of 13 dogs and 17 cats with a history of refractory CHF treated with subcutaneous (SC) furosemide were reviewed retrospectively. Administration of SC furosemide was offered as an alternative therapy when animals experienced an unsatisfactory clinical response to oral diuretics despite multiple dose adjustments and when, for this reason, pet owners were considering euthanasia. The satisfactory control of the animal’s breathing rate and effort and overall pet owner’s satisfaction were observed in all cases. Following administration of SC furosemide, the median survival time was 106 (95% CI: 22–154) days in dogs and 89 (95% CI: 35 to 749) days in cats. This study showed that furosemide administered subcutaneously appears to be an efficacious and feasible therapeutic option for providing control of the signs of cardiac congestion in both dogs and cats with a previous unsatisfactory response to oral diuresis.

## Simple Summary

Resistance to oral diuretics in the treatment of congestive heart failure (CHF) can be secondary to reduced intestinal drug absorption. A known therapeutic strategy to overcome this problem comprises the off-label subcutaneous (SC) administration of furosemide, although there are no available studies to support such an intervention in veterinary medicine. This retrospective study evaluated the feasibility of this route of administration in dogs and cats with a history of refractory CHF. Administration of SC furosemide was offered as an alternative therapy when animals experienced an unsatisfactory clinical response to oral diuretics despite multiple dose adjustments and when, for this reason, pet owners were considering euthanasia. The satisfactory control of the animal’s breathing rate and effort and overall pet owner’s satisfaction were observed in all cases. This study showed that furosemide administered subcutaneously appears to be an efficacious and feasible therapeutic option for providing control of the signs of cardiac congestion in both dogs and cats with a previous unsatisfactory response to oral diuresis.

## 1. Introduction

The expansion of extracellular fluid and increased renal sodium (Na) retention, which results from neurohormonal and hemodynamic fluctuations, are features of the pathophysiology of congestive heart failure (CHF). The above mechanisms lead to fluid accumulation within the interstitial space and/or in the body cavities, which causes a range of clinical signs such as tachypnea/dyspnea and abdominal distension [1,2]. Due to the crucial role of volume expansion and Na retention in causing congestion, diuretic therapy is an essential treatment for CHF, regardless of the underlying cardiac condition [1,2,3,4]. Therefore, high-ceiling loop diuretics are commonly used in human and veterinary cardiology due to their indisputable efficacy in relieving the signs of congestion [3,5].

In dogs and cats, the management of CHF has improved over the years, although the natural progression of the underlying primary cardiac disease requires loop diuretic dose adjustments and/or the further optimization of cardiac therapy to effectively control congestion. Indeed, many patients experience repeated hospital admissions due to relapses of congestive signs, requiring the administration of intravenous (IV) furosemide, which is the foundation of the treatment for acutely decompensated CHF both in human and veterinary medicine [6]. However, IV therapy can only be administered in a hospital setting and is typically used in the acute phase, making it an unsuitable option for chronic treatment at home, where oral medications represent the common standard of care. Nevertheless, the therapeutic efficacy of oral diuretics may decrease over time, requiring increasing daily doses of diuretics. In some cases, the clinical signs of congestion can become refractory despite maximal therapy (diuretic resistance) [1,2,7,8,9,10].

Diuretic resistance in dogs and cats can depend on several causes, including renal distal tubular hypertrophy (nephron remodeling), activation of the sympathetic nervous system and the renin-angiotensin aldosterone system, decreased diuretic delivery to the kidney (e.g., hypoalbuminemia) and/or secretion into the proximal convoluted tubule (e.g., chronic kidney disease), as well as reduced gastrointestinal absorption [1,2,7].

Subcutaneous (SC) administration of furosemide can potentially overcome the reduced gastrointestinal drug absorption. To date, there are only anecdotal reports of the utility of SC administration of furosemide in dogs and cats with CHF and suspected resistance to oral loop diuretics. Therefore, the first objective of this study was to determine the effectiveness of SC furosemide in controlling the signs of CHF in dogs and cats with refractory heart failure. Another objective was to describe the feasibility, compliance, and side effects of chronic SC furosemide therapy at home.

## 2. Materials and Methods

Clinical records for the dogs and cats in CHF that were treated with SC furosemide at The Ralph Veterinary Referral Centre between April 2020 and May 2024 were reviewed retrospectively. Subcutaneous administration of furosemide (Dimazon, MSD Animal Health UK Limited, Milton Keynes MK7 7AJ, UK) was offered as an alternative therapy when animals did not display a satisfactory clinical response to oral diuretics despite multiple dose adjustments and when, for this reason, their owners were considering euthanasia.

Data included patients’ details (species, breed, age, sex, neutering status, body weight), clinical information (clinical signs at presentation, diagnosis, therapy, sleeping respiratory rate at home [SRR]), and the number of relapses of CHF episodes.

In all cases, pet owners were instructed by clinical staff (nurses or clinicians) on how to correctly administer SC injections to their animals, indicating the interscapular region as the ideal site.

Three different timepoints were considered for this study. The first timepoint (T1) corresponded to the first onset of CHF. The second timepoint (T2) related to the onset of refractory CHF necessitating SC furosemide as a rescue therapy. Refractory CHF was diagnosed when affected pets required several hospitalizations despite alterations to their medical therapy, particularly diuretic dose adjustments, and when, according to their owners, clinical signs interfered with their pet’s daily activities and quality of life [8]. A minimum of two diuretic dose adjustments and the owner’s request to euthanize their pets were necessary to reach timepoint T2. The dose of SC furosemide at T2 was arbitrarily decided by the attending clinician, mainly based on the current oral dose of furosemide or the equivalent dose of torasemide.

The third timepoint (T3) corresponded to the patient’s death or, for patients still alive, the date at the end of data collection.

Assessment of quality of life (QoL) was based on the owner’s verbal feedback about their pet’s appetite, exercise, and grooming activity, as well as general pet-owner interaction.

Data distribution was assessed using the Shapiro-Wilk test and reported as either mean ± standard deviation (SD) for normally distributed data or median and 95% confidence interval (CI) for non-normally distributed data. The Kaplan-Meier method was used to estimate the median survival time of dogs and cats receiving SC furosemide.

Statistical analysis was performed using a commercially available software (MedCalc Software Ltd., Version 22.021, Ostend, Belgium).

## 3. Results

### 3.1. Animals

Our database query identified 30 client-owned pets (13 dogs and 17 cats) with a history of refractory CHF that underwent diuretic therapy with SC furosemide. The mean age of the dogs was 8.9 ± 2.1 years, while the mean age of the cats was 6.8 ± 4.8 years. The median body weight of the dogs was 10.0 Kg (95% CI: 7.3–27.4; range 7.4–27.4), while the mean body weight of the cats was 4.5 ± 0.9 Kg. A summary of the animals’ characteristics is reported in Table 1 and Table 2.

### 3.2. Diagnosis

Myxomatous mitral valve disease (MMVD) was the most common diagnosis in dogs (10 dogs; 76.9%), while the most common diagnosis in cats was hypertrophic cardiomyopathy (HCM) (six cats; 35.3%). All dogs and cats with left-sided CHF had evidence of left atrial enlargement on echocardiography and were reported to have tachypnea/dyspnea on clinical presentation. Dogs and cats with signs of right-sided CHF had abdominal distention and associated tachypnea. Sleeping respiratory rate (SRR) measured at home at T2 was elevated both in dogs (50.6 ± 16.1 breaths per minute) and in cats (53.2 ± 12.0 breaths per minute) [11].

A diagnosis of cardiogenic pulmonary edema was based on radiographic evidence of concomitant cardiomegaly, pulmonary vein congestion, and an interstitial–alveolar lung pattern. Pulmonary edema was observed in 10 dogs (76.9%) and 12 cats (70.5%). A diagnosis of pleural effusion was made in one dog (7.7%) and seven cats (41.2%), while ascites was observed in four dogs (30.8%) and one cat (5.9%). The diagnoses of pleural effusion and ascites were based on ultrasonographic evidence of cavitary effusions. Other signs of CHF are summarized in Table 1 and Table 2.

### 3.3. Diuretic Treatment and Clinical Outcome

At the time when oral diuretic therapy was substituted with injectable furosemide (T2), nine dogs (69.2%) were receiving oral furosemide (5.78 ± 2.36 mg/Kg/day), while four dogs (30.8%) were being treated with oral torasemide (0.31 ± 0.16 mg/Kg/day). At the same timepoint, twelve cats (70.6%) were having treatment with oral furosemide (5.35 ± 1.45 mg/Kg/day), while five cats (29.4%) were receiving oral torasemide (0.25 [95% CI: 0.20–0.50] mg/Kg/day). Additional therapeutic interventions are summarized in Table 1 and Table 2.

Oral diuretic therapy was substituted with SC furosemide injection at a median dose of 5.5 mg/Kg/day (95% CI: 4.0–7.6; range: 4.0–9.0) in dogs and a mean dose of 4.0 ± 1.34 mg/Kg/day in cats, divided into two equal administrations administered at 12 h intervals. In all cases there was a good clinical response, interpreted as satisfactory control of the animal’s breathing rate and effort and overall pet owner’s satisfaction.

All animal owners reported a noticeable improvement in their pet’s quality of life (QoL) between T2 and T3.

The median time from the first episode of CHF (T1) to an unsatisfactory clinical response to oral diuretic therapy (T2) was 71 days (95% CI: 37.5–177.4; range: 12–203) in dogs and 38 days (95% CI 15.1–134.9; range: 5–356) in cats.

**Figure 1:**
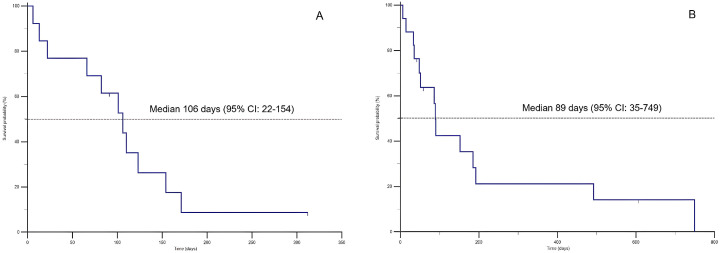
Kaplan-Meier survival curves for dogs (**A**) and cats (**B**) in refractory congestive heart failure (CHF) and receiving subcutaneous (SC) administration of furosemide. A tick on the curve represents a censored observation (animals still alive at the end of data collection).

The median survival time from T2 to T3 was 106 days (95% CI: 22–154) in dogs and 89 days (95% CI: 35 to 749) in cats. Sixteen animals (6 dogs and 10 cats) were euthanized due to a relapse of the signs of CHF and their negative impact on their QoL, although they displayed an initial positive clinical response following the initiation of SC furosemide. The remaining 14 animals died suddenly at home and, given the absence of other reported comorbidities, cardiac disease was considered as the cause of death in all these cases. At the end of the study, four pets (13.3%) were still alive. Survival data for dogs and cats receiving SC furosemide are reported in Table 3A,B and graphically represented by the Kaplan Meier curve in Figure 1A,B.

### 3.4. Adverse Reactions

Subcutaneous injections were well tolerated in most patients, with the exception of two dogs (15.3%) and three cats (17.6%) that experienced mild dermatologic adverse reactions at the site of injection (between the scapulae), characterized by irritation and scratching in the area followed by alopecia. One dog also developed a temporary soft skin lump, and two cats developed pyoderma which was successfully treated by the primary veterinarian with a short course of oral antibiotics (Table 3). It was unclear whether such adverse effects were predisposed by the injection site or the pet owner’s skill.

## 4. Discussion

The present study showed that SC furosemide appears effective, safe, and well tolerated, with minimal adverse effects in dogs and cats with refractory CHF.

The off-label use of SC furosemide is not a novelty; it has been adopted and advocated by veterinary cardiologists for many years, when oral administration is no longer effective in controlling the clinical signs of CHF, or where oral administration is logistically challenging for some pets, especially cats. This is due to its higher bioavailability compared with oral administration and improved compliance in some patients [3,12,13]. However, to the best of the authors’ knowledge, this is the first systematic evaluation of the effectiveness and feasibility of SC furosemide therapy in dogs and cats with refractory CHF.

A potential issue associated with daily SC injections is the ability of pet owners to correctly administer the drug via this route. For this reason, all pet owners received dedicated training from medical professionals, namely veterinary nurses/technicians and veterinarians. This training was also a valuable opportunity to assess the patient’s compliance and the ability of pet owners to perform this daily task before discharging the patients.

The dose of SC furosemide in this animal population was 5.5 mg/Kg/day (dogs) and 4 mg/Kg/day (cats), divided into two daily administrations. Such doses were arbitrarily decided by the attending clinician, often based on the previous oral dosage, and this proved to be effective in controlling the clinical signs in all patients.

An inevitable concern in selecting the parenteral administration of furosemide is the ability of pet owners to regularly continue this therapy at home and the pet’s tolerance for receiving twice-daily injections. This did not appear to be the case in this study, where SC furosemide was regularly administered in all dogs and cats without any concerns about the pet’s QoL.

Following the SC administration of furosemide, 7/13 (53.8%) dogs and 6/17 (35.3%) cats lived for more than three months, with three cats surviving more than one year (Figure 1). In our opinion, this can be interpreted as a positive outcome, considering that in all these patients the clinical signs of CHF could not be satisfactorily controlled using oral diuretics despite several dose adjustments, and that pet owners were already requesting euthanasia due to the compromised QoL of their animals.

Currently, there is no consensus in veterinary medicine about the criteria for selecting patients that can benefit from the SC administration of furosemide, although it seems intuitive to consider chronic parenteral administration in patients that have been successfully stabilized with IV furosemide following a relapse of CHF [3,12,13].

Subcutaneous administration of furosemide may also be considered when severe nausea and emesis, oral pain, dyspnea, and swallowing impairments are observed. In humans, the rate of absorption of SC furosemide depends on the vascularity of the area of injection and the solubility of the medication in the interstitial fluid. Furthermore, the rate of absorption following SC furosemide is sufficiently slow and consistent to provide a sustained effect similar to that of intramuscular injections [14].

In veterinary medicine, similar information is lacking except for a single study performed in healthy dogs where the maximum urine output was observed one hour after SC injection. The diuresis lasted longer (up to 2 h) compared to an IV injection, although the total urine volume produced during the 8 h study period was similar for both routes of administration, suggesting comparable bioavailability and effectiveness [15]. No information on SC furosemide bioavailability is currently reported.

Reduced gastrointestinal absorption is reported as one of the major causes of diuretic resistance in patients with right-sided CHF, likely caused by edema in the intestinal mucosa [16]. Interestingly, in this study, a beneficial effect of SC furosemide was also observed in dogs and cats with signs of left-sided CHF. This observation has also been reported in human medicine, where the chronic inflammation associated with CHF seems to affect not only the cardiovascular system but also the musculoskeletal, neuroendocrine, metabolic, and immune systems. This multisystemic disorder may be explained by the release of endotoxins (TNF-α) entering the circulation from the intestine in patients with both right- and left-sided CHF. Indeed, morphological and functional alterations in the large bowel are observed even in CHF patients without increased right atrial pressure, and this appears to be related to the sympathetic activation and vasoconstriction of intestinal vessels, inducing inadequate intestinal mucosal perfusion with secondary alteration of the gut microflora (gut–heart axis) [16,17,18,19]. Several preliminary studies have investigated this association in dogs, and it is not unrealistic to assume that gastrointestinal abnormalities (e.g., abnormal microbiota) might develop in dogs and cats with CHF, eventually leading to reduced diuretic responsiveness [20,21,22].

Most of the patients in this study appeared to tolerate the SC administration of furosemide without any noticeable side effects. Skin lesions represented the most commonly observed adverse reactions; they were characterized by local erythema, alopecia, pruritus, and a nodular lesion, as observed in 5/30 (16.6%) patients (Table 3). In three of these cases, skin reactions were minimal and resolved spontaneously simply by instructing the owners to frequently change the injection site. However, in the other two cases, oral antibiotic treatment was necessary to resolve the local pyoderma.

Skin reactions following SC furosemide injections are reported in a small number of human patients treated with SC furosemide [14], and in the veterinary literature similar lesions have been reported in one dog and one cat. Several potential explanations have been proposed, including mechanical trauma, poor sterility, and immune-mediated reactions [13,23]. The pH of injectable furosemide, especially the alkaline liquid formulation and the excipients contained in the preparation such as benzyl alcohol and/or sodium sulfite, may also be associated with these skin reactions [23]. Alkaline formulations are required to increase the solubility of furosemide, although alkalinity can also cause significant discomfort when furosemide is injected subcutaneously, which is one of the main reasons why furosemide is rarely used for SC administration in human medicine [24]. However, all our animals received the same alkaline (pH 9.0) solution and the same excipients (benzyl alcohol and sodium sulfite; Dimazon, MSD Animal Health UK Limited, Milton Keynes MK7 7AJ, UK), suggesting that the observed dermatological complications might also be associated with individual patient responses. Furthermore, it should be noted that skin lesions have also been reported in association with chronic oral furosemide therapy in a dog [25]. Injection site-associated sarcoma, previously reported in cats following the subcutaneous administration of vaccines, antibiotics, long-acting corticosteroids, and insulin, was not observed in this patient cohort [26].

Some important limitations, mainly associated with the retrospective nature and relatively small sample size of this study, need to be acknowledged. Firstly, the dose of furosemide at T2 was not standardized but arbitrarily chosen by the attending clinicians, as well as their decision on when to begin SC therapy. Furthermore, despite the satisfactory clinical response, no attempts were made to reduce the daily amount of furosemide to the minimal effective dose. In people, for example, when a patient is switched from intravenous to oral loop diuretics, the dose of furosemide should be doubled due to so-called “absorption-limited kinetics” [27]. Indeed, the furosemide half-life is short, but its duration of action is longer when administered orally as its gastrointestinal absorption may be slower than its elimination. Therefore, it would be realistic to assume that, in the same patient, the parenteral dose of furosemide required should be lower than the previous oral dose.

Secondly, the dogs and cats in this study were affected by different cardiovascular conditions characterized by differing disease progression, which has likely affected the survival of the various individuals.

Thirdly, an unsatisfactory response to oral diuretics was mostly based on subjective assessment, which was mainly a composite of elevated SRR and reduced QoL reported by the pet owners [11]. This may appear in contrast with some recommended guidelines for the diagnosis and management of canine myxomatous mitral valve disease and feline cardiomyopathies, where refractory heart failure is characterized by a daily furosemide dose equal to or higher than the 8 mg/Kg/day in dogs or higher than the 6 mg/Kg/day in cats (or the equivalent dose of torasemide) required to control the signs of cardiac congestion [3,28]. However, the definition of refractory heart failure may also take into consideration additional parameters, such as the number of hospitalizations, the number of modified therapies, the addition of anti-arrhythmic agents, renal function, presence of comorbidities, weight loss, and reduced QoL [8]. Assessment of QoL in these dogs and cats was based on a purely subjective assessment by pet owners rather than a semi-objective assessment, such as the CATCH and FETCH scoring system [29,30].

Finally, it should be noted that multiple factors prevent this research from offering definitive answers regarding the use of SC furosemide in the clinical setting, and further investigations should be conducted to determine the appropriate timing for its use and the dosages administered, as well as the functional and structural changes at the cardiovascular and renal levels.

Based on these considerations, it would be desirable to have a consensus amongst veterinarians to subjectively describe diuretic resistance in dogs and cats in order to provide individualized treatment plans based on diuretic responsiveness, prevention of congestion, and decreased adverse effects [2,31].

## 5. Conclusions

Subcutaneous furosemide administration appears to be a feasible alternative intervention for providing efficacious palliative care at home for dogs and cats with CHF and an unsatisfactory response to oral diuresis. This appears to be an ideal option for patients where diuretic resistance is secondary to reduced gastrointestinal absorption, which would also impact the efficacy of other oral diuretics such as thiazides. However, the risk of adverse reactions, a pet’s temperament, and the willingness of pet owners to perform injections on their pet at home, as some owners may be unable or unwilling to comply (e.g., needle-phobia, also known as trypanophobia), may negatively affect this therapeutic option.

## Figures and Tables

**Table 1:** Patients’ signalment, diagnosis, clinical signs, and oral loop diuretic dose in 13 dogs.

Subject	Breed	Sex	Age (Years)	Weight (Kg)	Diagnosis	CHF	SRR T2	Diuretic T2	Oral Diuretic Dose (mg/Kg/day)	Other Therapies
Dog 1	Beagle	FN	10	14.0	MMVD	PE	80	Torasemide	0.4	Benazepril, Spironolactone, Pimobendan, Digoxin
Dog 2	Chihuahua	M	8	7.4	MMVD	PE	68	Furosemide	3.0	Benazepril, Spironolactone, Pimobendan
Dog 3	Chihuahua	F	9	4.9	MMVD	PE	72	Furosemide	6.0	Benazepril, Spironolactone, Pimobendan
Dog 4	JRT	MN	10	8.1	MMVD	PE	45	Furosemide	7.25	Benazepril, Spironolactone, Pimobendan, Codeine
Dog 5	Labrador	MN	11	33.2	AVDys + AF	PE; Ascites	60	Furosemide	3.6	Benazepril, Spironolactone, Pimobendan, Sotalol
Dog 6	Labrador	FN	11	37.7	MMVD + AF	Ascites	30	Furosemide	8.0	Benazepril, Spironolactone, Pimobendan, Digoxin, Diltiazem
Dog 7	CKCS	FN	6	7.3	MMVD	PE	32	Furosemide	8.0	Benazepril, Spironolactone, Pimobendan
Dog 8	DDB	FN	8	49.3	TVDys + AF	PLE; Ascites	40	Furosemide	8.2	Pimobendan, Spironolactone, Digoxin, Diltiazem
Dog 9	Bulldog	MN	6	22.3	ARVC	Ascites	44	Furosemide	6.0	Pimobendan, Spironolactone, Amiodarone
Dog 10	CKCS	MN	8	9.0	MMVD	PE	60	Torasemide	0.2	Pimobendan, Benazepril, Spironolactone
Dog 11	Beagle	FN	12	10.0	MMVD + PH	PE	50	Torasemide	0.15	Pimobendan, Benazepril, Spironolactone
Dog 12	Chihuahua	MN	6	3.2	MMVD	PE	45	Torasemide	0.5	Pimobendan, Benazepril, Spironolactone, Digoxin
Dog 13	CKCS	MN	11	11.3	MMVD	PE	32	Furosemide	2.0	Pimobendan, Benazepril, Spironolactone

CHF: congestive heart failure; SRR: sleeping respiratory rate at home; M: male; F: female; MN: male neutered; FN: female neutered; MMVD: myxomatous mitral valve disease; AVDys: bilateral atrioventricular valve dysplasia; TVDys: tricuspid valve dysplasia; ARVC: arrhythmogenic right ventricular cardiomyopathy; AF; atrial fibrillation; PE: pulmonary edema; PLE: pleural effusion;PH: pulmonary hypertension; JRT: Jack Russell Terrier; CKCS: Cavalier King Charles Spaniel, DDB: Dogue De Bordeaux.

**Table 2:** Patients’ signalment, diagnosis, clinical signs, and oral loop diuretic dose in 17 cats.

Subject	Breed	Sex	Age (Years)	Weight (Kg)	Diagnosis	CHF	SRR T2	Oral Diuretic T2	Diuretic Dose (mg/Kg/day)	Other Therapies
Cat 1	DSH	MN	7	5.2	MVDys	PE	49	Torasemide	0.2	Spironolactone, Clopidogrel
Cat 2	Bengal	FN	3	3.5	AS	PE	68	Furosemide	6.4	Clopidogrel
Cat 3	Sphynx	MN	4	3.5	HCM + SAM	PE	80	Torasemide	0.25	None
Cat 4	Scottish	MN	1	3.9	HCM	PE	50	Torasemide	0.25	None
Cat 5	Siberian	MN	8	3.9	ESC	PE	52	Furosemide	4.0	Clopidogrel
Cat 6	DSH	MN	11	6.0	ESC	PE	64	Furosemide	6.6	Pimobendan, Clopidogrel, Sotalol
Cat 7	DSH	MN	1	5.6	HCM	PE, PCE	60	Furosemide	3.6	Clopidogrel
Cat 8	BSH	FN	1	3.1	HCM	PE	44	Furosemide	4.0	Clopidogrel
Cat 9	DSH	MN	4	4.0	VSD	PE	48	Torasemide	0.25	Clopidogrel
Cat 10	DSH	MN	10	6.0	HCM	PLE	60	Torasemide	0.5	Clopidogrel
Cat 11	Scottish	MN	1	5.0	MVDys + SAM	PE	45	Furosemide	5.0	Clopidogrel
Cat 12	BSH	MN	5	5.2	TVDys + AF	PLE, Ascites	44	Furosemide	8.0	Spironolactone, Clopidogrel
Cat 13	Sphynx	FN	12	3.4	RCM	PLE	72	Furosemide	6.0	Clopidogrel
Cat 14	DSH	MN	15	3.5	RCM	PLE	40	Furosemide	5.7	Pimobendan, Clopidogrel
Cat 15	DSH	MN	14	4.8	RCM + AF	PE, PLE	38	Furosemide	6.0	Clopidogrel
Cat 16	DSH	MN	7	4.6	ESC	PLE, PE	48	Furosemide	6.0	Pimobendan, Clopidogrel
Cat 17	DSH	MN	11	5.0	HCM + SAM	PLE	44	Furosemide	3.0	Clopidogrel, Benazepril, Spironolactone

CHF: congestive heart failure; SRR: sleeping respiratory rate at home; MN: male neutered; FN: female neutered; MVDys: mitral valve dysplasia; AS: aortic stenosis; HCM: hypertrophic cardiomyopathy; SAM: systolic anterior motion of the mitral valve; ESC: end-stage cardiomyopathy; VSD: ventricular septal defect; TVDys: tricuspid valve dysplasia; AF: atrial fibrillation; RCM: restrictive cardiomyopathy; PE: pulmonary edema; PLE: pleural effusion; PCE: pericardial effusion; DSH: Domestic short hair; BSH: British short hair.

**Table 3:** Summary of the clinical progression in 13 dogs (A) and 17 cats (B) diagnosed with congestive heart failure (CHF), described as time from diagnosis (T1) to development of diuretic resistance (T2). The period T2–T3 describes the survival time associated with the administration of subcutaneous (SC) furosemide. Furosemide dose T3 is reported as a total daily dose (mg/Kg/day), which was divided into two equal doses administered at 12 h intervals. Adverse reactions, when present, following subcutaneous furosemide injections are also reported.

**A**					
**Patients**	**T1–T2 (Days)**	**T2–T3 (Days)**	**SC Furosemide Dose T3 (mg/Kg/day)**	**Adverse Reactions (T2–T3)**	**Cause of Death**
Dog 1	177	106	8.4	Mild skin reaction (alopecia/irritation)	Euthanasia
Dog 2	71	6	4.0		Sudden death
Dog 3	36	66	6.0		Sudden death
Dog 4	32	22	7.8		Euthanasia
Dog 5	180	82	4.0		Euthanasia
Dog 6	148	123	7.4		Euthanasia
Dog 7	39	13	9.0		Sudden death
Dog 8	163	171	6.6		Sudden death
Dog 9	65	110	4.0		Sudden death
Dog 10	178	91	5.5	Moderate skin reaction (scratching, alopecia, temporary lump)	Alive
Dog 11	203	101	4.0		Euthanasia
Dog 12	41	312	4.4		Alive
Dog 13	12	154	4.4		Euthanasia
**B**					
**Patients**	**T1–T2 (Days)**	**T2–T3 (Days)**	**SC Furosemide Dose T3 (mg/Kg/day)**	**Adverse Reactions (T2–T3)**	**Cause of Death**
Cat 1	139	492	4.0		Euthanasia
Cat 2	15	6	6.4		Sudden death
Cat 3	35	749	4.0		Euthanasia
Cat 4	19	48	6.0		Sudden death
Cat 5	5	192	6.0	Moderate skin reaction (scratching, alopecia, pyoderma)	Sudden death
Cat 6	129	90	7.5		Euthanasia
Cat 7	31	51	4.6		Euthanasia
Cat 8	38	35	4.0		Euthanasia
Cat 9	5	185	4.0		Sudden death
Cat 10	130	152	5.0	Moderate skin reaction (scratching, alopecia and pyoderma)	Euthanasia
Cat 11	9	504	6.0		Euthanasia
Cat 12	135	89	4.0		Sudden death
Cat 13	13	14	5.6		Euthanasia
Cat 14	155	33	4.0		Euthanasia
Cat 15	356	42	4.0	Mild skin irritation; scratching	Alive
Cat 16	72	58	3.4		Alive
Cat 17	343	86	2.0		Euthanasia

## Data Availability

Data presented in this study are available on request from the corresponding author. Data are not publicly available due to patient data protection.

## References

[1] Mullens W., Damman K., Harjola V.P., Mebazaa A., Brunner-La Rocca H.P., Martens P., Testani J.M., Tang W.H.W., Orso F., Rossignol P. (2019). The use of diuretics in heart failure with congestion—A position statement from the Heart Failure Association of the European Society of Cardiology. Eur. J. Heart Fail..

[2] Oyama M.A., Adin D. (2023). Toward quantification of loop diuretic responsiveness for congestive heart failure. J. Vet. Intern. Med..

[3] Keene B.W., Atkins C.E., Bonagura J.D., Fox P.R., Haggstrom J., Fuentes V.L., Oyama M.A., Rush J.E., Stepien R., Uechi M. (2019). ACVIM consensus guidelines for the diagnosis and treatment of myxomatous mitral valve disease in dogs. J. Vet. Intern. Med..

[4] Poissonnier C., Ghazal S., Passavin P., Alvarado M.P., Lefort S., Trehiou-Sechi E., Saponaro V., Barbarino A., Delle Cave J., Marchal C.R. (2020). Tolerance of torasemide in cats with congestive heart failure: A retrospective study on 21 cases (2016–2019). BMC Vet. Res..

[5] Buggey J., Mentz R.J., Pitt B., Eisenstein E.L., Anstrom K.J., Velazquez E.J., O’Connor C.M. (2015). A reappraisal of loop diuretic choice in heart failure patients. Am. Heart J..

[6] Owen D.R., MacAllister R., Sofat R. (2015). Intravenous Furosemide for Acute Decompensated Congestive Heart Failure: What Is the Evidence?. Clin. Pharmacol. Ther..

[7] Adin D., Atkins C., Papich M.G. (2018). Pharmacodynamic assessment of diuretic efficacy and braking in a furosemide continuous infusion model. J. Vet. Cardiol..

[8] Behnoush A.H., Khalaji A., Naderi N., Ashraf H., von Haehling S. (2023). ACC/AHA/HFSA 2022 and ESC 2021 guidelines on heart failure comparison. ESC Heart Fail..

[9] Ellison D.H., Felker G.M. (2017). Diuretic Treatment in Heart Failure. N. Engl. J. Med..

[10] Hoorn E.J., Ellison D.H. (2017). Diuretic Resistance. Am. J. Kidney Dis..

[11] Porciello F., Rishniw M., Ljungvall I., Ferasin L., Haggstrom J., Ohad D.G. (2016). Sleeping and resting respiratory rates in dogs and cats with medically-controlled left-sided congestive heart failure. Vet. J..

[12] Bonagura J.D.T., Twedt D.C. (2009). Management of Heart Failure in Dogs.

[13] Scruggs S.M., Rishniw M. (2013). Dermatologic adverse effect of subcutaneous furosemide administration in a dog. J. Vet. Intern. Med..

[14] Zacharias H., Raw J., Nunn A., Parsons S., Johnson M. (2011). Is there a role for subcutaneous furosemide in the community and hospice management of end-stage heart failure?. Palliat. Med..

[15] Harada K., Ukai Y., Kanakubo K., Yamano S., Lee J., Kurosawa T.A., Uechi M. (2015). Comparison of the diuretic effect of furosemide by different methods of administration in healthy dogs. J. Vet. Emerg. Crit. Care.

[16] Sundaram V., Fang J.C. (2016). Gastrointestinal and Liver Issues in Heart Failure. Circulation.

[17] Desai D., Desai A., Jamil A., Csendes D., Gutlapalli S.D., Prakash K., Swarnakari K.M., Bai M., Manoharan M.P., Raja R. (2023). Re-defining the Gut Heart Axis: A Systematic Review of the Literature on the Role of Gut Microbial Dysbiosis in Patients with Heart Failure. Cureus.

[18] Sandek A., Bauditz J., Swidsinski A., Buhner S., Weber-Eibel J., von Haehling S., Schroedl W., Karhausen T., Doehner W., Rauchhaus M. (2007). Altered intestinal function in patients with chronic heart failure. J. Am. Coll. Cardiol..

[19] Li Q., Larouche-Lebel E., Loughran K.A., Huh T.P., Suchodolski J.S., Oyama M.A. (2021). Metabolomics analysis reveals deranged energy metabolism and amino acid metabolic reprogramming in dogs with myxomatous mitral valve disease. J. Am. Heart Assoc..

[20] Araki R., Iwanaga K., Ueda K., Isaka M. (2021). Intestinal Complication with Myxomatous Mitral Valve Diseases in Chihuahuas. Front. Vet. Sci..

[21] Karlin E.T., Rush J.E., Freeman L.M. (2019). A pilot study investigating circulating trimethylamine N-oxide and its precursors in dogs with degenerative mitral valve disease with or without congestive heart failure. J. Vet. Intern. Med..

[22] Li Q., Larouche-Lebel E., Loughran K.A., Huh T.P., Suchodolski J.S., Oyama M.A. (2021). Gut Dysbiosis and Its Associations with Gut Microbiota-Derived Metabolites in Dogs with Myxomatous Mitral Valve Disease. mSystems.

[23] Mazzoldi C., Aspidi F., Romito G. (2023). Dermatologic adverse effect of subcutaneous furosemide administration in a cat. Open Vet. J..

[24] Sica D.A., Muntendam P., Myers R.L., Ter Maaten J.M., Sale M.E., de Boer R.A., Pitt B. (2018). Subcutaneous Furosemide in Heart Failure: Pharmacokinetic Characteristics of a Newly Buffered Solution. JACC Basic Transl. Sci..

[25] Ochoa P.G., Arribas M.T., Mena J.M., Perez M.G. (2006). Cutaneous adverse reaction to furosemide treatment: New clinical findings. Can. Vet. J..

[26] Ladlow J. (2013). Injection site-associated sarcoma in the cat: Treatment recommendations and results to date. J. Feline Med. Surg..

[27] Ellison D.H. (2019). Clinical Pharmacology in Diuretic Use. Clin. J. Am. Soc. Nephrol..

[28] Luis Fuentes V., Abbott J., Chetboul V., Cote E., Fox P.R., Haggstrom J., Kittleson M.D., Schober K., Stern J.A. (2020). ACVIM consensus statement guidelines for the classification, diagnosis, and management of cardiomyopathies in cats. J. Vet. Intern. Med..

[29] Freeman L.M., Rush J.E., Farabaugh A.E., Must A. (2005). Development and evaluation of a questionnaire for assessing health-related quality of life in dogs with cardiac disease. J. Am. Vet. Med. Assoc..

[30] Freeman L.M., Rush J.E., Oyama M.A., MacDonald K.A., Cunningham S.M., Bulmer B., MacGregor J.M., Laste N.J., Malakoff R.L., Hall D.J. (2012). Development and evaluation of a questionnaire for assessment of health-related quality of life in cats with cardiac disease. J. Am. Vet. Med. Assoc..

[31] Damman K., Ter Maaten J.M., Coster J.E., Krikken J.A., van Deursen V.M., Krijnen H.K., Hofman M., Nieuwland W., van Veldhuisen D.J., Voors A.A. (2020). Clinical importance of urinary sodium excretion in acute heart failure. Eur. J. Heart Fail..

